# Selections and Abstracts

**Published:** 1902-11

**Authors:** 


### SELECTIONS AND ABSTRACTS.

### Quack Medicine.

The proper consideration of this subject presupposes an official, detailed analysis which is not available in this country and which is one of the duties of the national body of physicians or of one of the government medical services, and the execution of which should be urged for the near future. From legal reasons, it is obviously impossible to deal specifically with the subject, from present occasional unofficial analyses or supposititious information as to the composition of popular remedies.

Beside the ordinary quack nostrums, we must remember that even an ethical proprietary compound may be similarly misused, and that many secret remedies otherwise introduced to the midical and pharmacal professions in an apparently ethical way are really intended for lay use, and that some manufacturers appeal to the medical profession through the ordinary channels for support, while advertising to the laity in the popular press. Individual druggists also prepare imitations of various nostrums, for prescription over the counter, and it may be pertinent to inquire how long the medical profession will continue its patronage of wholesale or retail pharmacists guilty of this form of double-dealing.

The enormous vogue of quack medicines is explained partly by the popular love of mysticism and humbug, partly by inaccessibility in terms of distance or expense of regular medical advice, partly by failure on the part of the laity to realize the paramount importance of accurate diagnosis as a basis of therapeutic measures. The profession is largely responsible for these conditions. When the physician discards placebos and suggestion, ceases to appeal to the confidence of the patient, and honestly states that his diagnosis is in doubt, not that he hopes to avert this or that "threatened" disease, and places the healing art squarely before his clientele as based upon two factors, the application of scientific principles and of empiric knowledge in the absence of full understanding of cause and effect, then, and not till then, may we expect the popular conception of medical mysticism to be supplanted by a spirit of loyal and intelligent coöperation.

The second obstacle must be met by professional organization which shall provide for the remote inhabitant, and for the large class which lies between comparative affluence and avowed or implied pauperism, and which must now depend largely upon the charlatan or upon inefficient and unjust lay schemes of insurance against injury and disease.

The laity will never learn the value of disagnosis—which includes far more than the naming of disease—while physicians in good standing prescribe without adequate examination, judge of anemia by the complexion and diagnose malaria where neither the parasite nor its means of conveyance is indigenous, or allow diabetes and nephritis to advance nearly to a fatal issue before it occurs to them to investigate the urine.

Most proprietary remedies are based on the same drugs as are employed by the regular profession. The dangers in their use are:

1. The probability of neglecting indications that do not appear from a superficial and unskilled diagnosis.

2. The impossibility of pushing powerful drugs in emergent cases without close supervision and, in general, the impossibility of applying a ready-made formula to any particular case with more than a mathematic probability of accomplishing the desired end.

3. The toxic action of the medicine itself. While acute poisoning is usually guarded against, cumulative action often results in chronic poisoning, or even syrups or other components, which can scarcely be considered toxic in the ordinary sense, may produce derangements of digestion, chronic inflammatory changes and other complications.

The first two objections must be understood as applying to all of the various kinds of nostrums to be discussed, while the mere citation of active drugs will suffice to suggest their well-known toxic action, which can not be considered in detail in a paper of moderate length.

1. *Bitters.* Most of the simple aromatic bitters are used in various combinations, usually with considerable amounts of alcohol, even when the opposite is claimed, as stomachics, spring-of-the-year medicines, liquor substitutes for temperance advocates, supporters during such critical periods as menstruation, pregnancy, lactation, the menopause, or during periods of strain and overwork. The bitters are notoriously inefficient and, therefore, comparatively harmless, though their protracted use may cause gastro-enteric catarrh or clonic or tonic overstimulation of peristalsis. Considered as alcoholics, these medicines sometimes act beneficially in inducing a moderate indulgence in those who would otherwise drink to excess, but, on the other hand, they may lead to alcoholism on the part of those not otherwise tempted. They can be of value only as temporary spurs to digestive secretion and motility, and possibly to absorption.

2. *Dyspepsia Cures.* While the bitters were, and are still, largely advertised for this purpose, animal and vegetable digestive ferments are now prepared for lay use, on a considerable scale. As the ferments of the body are almost the last factors to yield to organic or functional disturbance, such remedies are rarely indicated, though they are employed almost as much and as irrationally by the medical profession as by the laity. Such drugs fail to strike at the root of the trouble and they weaken the natural and spontaneous digestive strength of the body, by supplying an artificial and transient substitute.

3. *Cough Syrups, Consumption and Asthma Cures, Etc.* Many of these are opiates, containing morphine or codeine or other preparations of opium, or else some drying and sedative drug like atropine. It should be remembered, however, that they are no worse than the Bellevue Cough Mixture and various favorite formulæ and ethical proprietary syrups, used by the regular profession. Some few are comparatively innocuous or even excellent preparations of tar, balsams and other stimulant expectorants. The croup cures usually contain ipecac, often with tartar emetic, and some of the more recent contain apomorphine.

Asthma nostrums are often sold in pairs, liquids for internal use and powders or papers for fumigation. The former contain lobelia, grindelia, hyoscyamus, stramonium, etc.; the latter alkaline nitrites, stramonium leaves, etc. The more modern asthma quacks employ nitroglycerine, amyl nitrite, etc. As *Asthma*, even as used by the medical profession, includes chronic bronchitis and emphysema and is the expression of the most diverse constitutional states, such as renal and cardiac failure, perversions of nutrition, organic central nervous lesions, peripheral nervous reflex from the ovaries, cervix, uteri, rectum, gall-bladder, etc., it is obvious that the direct treatment of the spasm of the bronchioles, however well applied, is a small part of the radical treatment of the disease.

4. *Catarrh Cures.* The old-fashioned snuffs are now almost obsolete. The nasal douche, unless very carefully employed, is apt to injure the Schneiderian membrane, even with a saline solution. Hence, even the quacks have mainly abandoned it and now use sprays of various kinds, chiefly watery solutions of alkalies, mild antiseptics and astringents, and solutions in mineral oil of menthol, eucalyptol, camphor, etc. Many of the hydrocarbon bases are imperfectly refined, but, on the whole, it is mainly in the failure of nice discrimination that the proprietary sprays differ from those used by most regular practitioners.

Inhalations, whether used with a tube or intended to be breathed from the hand or from hot water, or whether pungent drugs like cubebs are burned in a cigarette, are very similar to similar preparations of ethical standing. In all such medicaments, however, we need to guard against excessive use, especially when carbolic acid and iodine and astringents are contained.

5. *Soothing Syrups.* These are practically always syrupy opiates or occasionally milder depressants, such as bromids. Even the lay press has been instrumental in warning against their use, and only the most ignorant or depraved mothers and nurses now employ them. Their baneful effect is threefold—immediately against the nervous centers, immediately against the digestive organs, and ultimately tending to chronic nervous and nutritive weakness, which may be lifelong.

6. *Rheumatism Cures.* The older representatives of this class contained alkalies, rhus toxicodendron, phytolacca, iodids, sarsaparilla, etc. At one time or another almost every cheap *alterative* has been thus employed. The new formulæ contain salicylates, variously combined. Most vegetable alteratives are comparatively inert, but phytolacca, iodids and salicylates are toxic if taken continuously. Rheumatism, as diagnosed by the laity, includes true chronic rheumatism, myalgia, neuralgia, *neuritis*, pain due to *organic disease, tertiary syphilis, tubercular and surgical diseases of bones and joints*, those italicized being especially likely to develop to a serious stage if neglected.

7. *Cures for Syphilis and Blood Disorders.* These are usually advertised so as to include chronic rheumatism, acne and the most diverse conditions. Mercury is seldom included in the more modern nostrums, on account of the impossibility of controlling its ingestion without elaborate warnings that would alarm prospective users. Iodids are usually contained in these medicines, even the sarsaparillas and "purely vegetable" compounds being said to contain them in most instances.

8. *Skin Cures.* Those for external use consist mainly of sulphur, in a mild antiseptic wash, and are probably harmless, though of little value except against acne. The internal remedies are mainly based on arsenic, and the danger both of systemic poisoning, including the setting up of a chronic nephritis, and of producing ultimately a thick, crackly hide, amounts almost to a certainty.

9. *General Tonics.* These comprise iron, manganese, strychnine, quinine, so-called beef, iron and wine, some of which are genuine, and some of which contain cheap mixtures of iron, alcohol and proteid; alcoholic bitters, variously altered, cod liver oil emulsions, malt preparations, which may be nothing but beer on the one hand or molasses on the other; and preparations of hypophosphites or phosphates. It will be seen that very few of these are apt to produce serious toxic symptoms but that minor disturbances of digestion may be caused.

10. *Headache Remedies.* While the older ones were chiefly saline cathartics, the modern ones are nervines, divided into two classes, powders containing acetanilid or some similar coal-tar derivative, and, occasionally, cocain or some other anodyne alkaloid; and, secondly, effervescent saline preparations of bromid, caffein and phosphates. The latter, being in most instances, physiologically incompatible, produce little trouble except from the encouragement of pernicious habits, although, if used excessively, one or other of the active ingredients may produce characteristic chronic symptoms. Several classic cases of acetanilid poisoning have been reported from the use of headache powders and possibly some inexplicable cases of sudden heart-failure may be due to this habit.

11. *Cures for the Alcohol and Other Drug Habits.* The old-fashioned substitutes for alcohol have already been considered. The modern extraprofessional cures are chiefly carried on at institutions, and it is said depend upon the use of strychnine or atropine as a stimulant and the simultaneous injection or administration in other form of apomorphine, whenever liquor is allowed as a test. The patient soon becomes disgusted and is convinced that he is unable to tolerate alcohol in any form. Various gums and sialagogues are employed to take the place of the local stimulation of tobacco, while the inner bark of the tulip tree is said, in some unexplained way, to produce a distaste for tobacco in any form. The essential element in any successful treatment of a habit is to secure the patient's co-operation, and it is a question whether the end does not justify the means. At any rate, it is absurd to suppose that the few cases of insanity which have occurred in the inmates of unethical institutions for the cure of drug habits are due to the means employed, when we consider the liability of these patients to mental alienation. But it is unfortunate that hospitals under control of the regular medical profession cannot be established for the care of drug habitués.

12. *Cathartics.* Some of these are advertised as liver remedies, others are or were in vogue as headache cures, but the majority are avowedly cathartics. Except the liver pills, few contain mercurials, but are made up after the model of an "A. S. & B." pill, with many modifications. Many patients buy tablets primarily intended for physicians' prescriptions. The teas and powders are composed mainly of compound licorice powder, senna, and similar mild laxatives. Of late years, cascara has become the favorite laxative both of the quack and the regular physician. Probably for occasional use most of the cathartics are allowable, but for any chronic state the utmost caution is necessary, and the use of atropine or any other drug that has any decided constitutional effect is undesirable.

13. *Liniments, Salves and Plasters.* The absorption of morphine, cantharides, atropine, etc., must be borne in mind, but most such preparations are comparatively harmless.

14. *Hair Tonics, Dyes and Bleaches.* The older hair tonics were diluted alcoholic solutions of quinine or some other supposedly local stimulant. The recent ones usually contain jaborandi. The dyes contain lead, silver, or cyanogen compounds and may cause systemic poisoning, either by direct absorption or by being gradually washed out of the hair by beverages. The effect upon the hair is slowly toxic. Bleaches formerly depended mainly on borax and alkalies, at present upon hydrogen peroxide. While systemically harmless, the hair loses its luster and premature greyness is caused.

15. *Pain-Killers.* Those for internal use contain an opiate, chloroform or mydriatic or cardiac depressant anodyne. Those for local use, as dental wax or gum, "balms," etc., for application to the external surface depend upon the presence of aconite, veratrum, cocain, gelsemium and other dangerously depressing drugs.

16. *Gonorrhea Cures.* Most of these, whether for use by injection, bougie or internal use, are identical with those used by the regular physician, with the exception of the later remedies. The danger of complications, especially from any injection sufficiently strong to warrant the claim of a rapid cure, the danger of self-infection, especially in the eye, and of infection of others from relying upon a sweeping promise of cure, need no discussion.

17. *Monthly Regulators.* Practically all of these owe their sale to the desire to produce an abortion, and, if not, an amenorrhea due to tuberculosis, chlorosis or other constitutional disease, or to intrinsic pelvic disease, requires the most careful treatment. In the majority of cases we must consider first the acute toxic action of tansy, pennyroyal, parsley, ergot, etc., on the system at large; secondly, the effect of aloin and the ordinary oxytocics in producing pelvic congestion; thirdly, the result, in shock and danger of sepsis, of the abortion itself.

18. *Leucorrhea Injections, Suppositories, etc.* These contain boric acid, tannin, as such, or in some vegetable product, hydrastis, aluminum, zinc, etc. Their local effect may inflame the parts, but they seldom produce serious general symptoms.

19. *Sexual Tonics.* This group of drugs, familiar to all through the "before and after" illustrations, consist of phosphorus, damiana, strychnine, quinine, cantharides, etc. The danger from their use includes the direct toxic action and the incentive to sexual excess.

20. Local sexual stimulants may consist of any simple ointment to be applied with friction or, for males, of suction pumps to produce engorgement. All such measures are practically modes of masturbation.

21. Instruments and injections or suppositories for the prevention of conception exercise their greatest harm along moral lines. They must be admitted to have had a beneficial effect in diminishing the frequency of interrupted coitus. The chemicals employed to kill spermatozoa may exert a toxic action, but even corrosive sublimate need not be in sufficient strength to cause appreciable absorption, unless used immoderately often.

22. *Bust Developers.* These are either mechanic—suction devices similar to breast pumps—or depend upon increasing the local blood supply by the friction used in applying any ointment or lotion, or upon the increase of secretory activity by iodids, etc. They may possibly predispose to organic disease of the breasts by traumatism, and certain drugs like iodids may be absorbed.

These last few classes of quack medicines and applications, aside from their direct physical and moral dangers, are serving to educate the youth of the land in sexual matters from the meanest and most depraved standpoint.

23. *Worm Medicines.* The common form is a santonin confection for children. Intestinal parasites are by no means so common as supposed by the laity, and these drugs, all more or less toxic, are given too freely and often unnecessarily.

24. *Eye Washes, Cataract Cures, etc.* The former contain salt, zinc, boric acid, etc. Lead and alum are somewhat dangerous, the milder astringents merely irritating and often ineffective. Cataract cures depend either upon the fictitious increase of vision by mydriatics or upon counterirritation, as by the volatile oil of mustard. The constitutional danger of the former and the local damage possible under the latter treatment are obvious. Here may be mentioned the pumps intended to remedy refractive errors by improving the shape of the eyeball. Such instruments are dangerous by reason of the possibility of internal hemorrhage and retinal detachment, and are of course useless to effect a permanent correction.

25. Finally, may be mentioned the absolute frauds, for local or systemic use, whether purporting to be drugs, sources of oxygen, electricity, vital energy, antiseptic radiation or what-not. These are, of course, as harmless as they are useless.—*A. L. Benedict, A.M., M.D., in Medical Times.*

---

### CAN ONE BECOME IMMUNE TO THE HONEY BEE'S STING?

As we are reading so much lately, both in medical and other literature, concerning the honey bee, and the action of its poison or sting, *apis mel*, as an important therapeutic agent, especially in local congestions, it may not be out of the way to consider the question of immunity arising from a constant introduction into the system of the poison which a bee injects by means of its sting into the person accustomed to handle or work amongst them. In other words, can a person so employed become immune to the virus of the honey bee?

I have no "control experiments" to give me data in regard to the matter, but will cite some of my personal experiences with quotations from prominent writers on bee literature, to try and show that one may become immune.

For a number of years, I used to fear and dread the honey bee, or in fact any other sting-bearing insect. A few years ago, becoming interested in the production of honey, and the life and habits of the honey bee, I determined to try, at least, to lay aside my objections to the stinging qualities and pursue the study of these interesting insects as a pastime and recreation, which at times becomes very acceptable to all professional men to keep them from tiring too much of the humdrum ruts of their lives.

When I first took up apiculture, I was frequently stung. If my veil was improperly adjusted or had the slightest hole in it, a bee was sure to find it out and entering, upon his departure, if such was possible, would leave his gentle reminder in some part of my head. If my gloves did not properly protect me, he would find there a vulnerable spot, or if I left my trousers unfastened at the bottom or wore low shoes, the little fellow knew it at once and delighted in finding some particular spot to leave his unfriendly calling card. The part thus stung would at once begin to burn, pain and become swollen, the pain being so acute that it is impossible to describe it, and must be experienced to be fully appreciated.

I noticed, however, as time went on, that the stinging did not bother me so much, neither did the parts swell in proportion. I attributed it to the fact that I was beginning to understand how to handle bees in a more scientific and skillful manner and congratulated myself on the matter.

In the second year of my experience I came into possession of a hive of bees that needed to be transferred to a modern hive, and as it necessitated the entire pulling apart of the original hive, with its 25,000 bees (for that is the usual number in a thrifty hive, colony or skep), I undertook the task with fear and trembling, knowing full well that when once the work was started there would be no possible chance for cessation until they were all safely hived in their new home.

Unquestionably the idea of the number of stings I would receive deterred the matter for several days, as I well knew that with that number of bees flying around, the old and young bee ready to avenge the seeming injury done to them, and as mad as "March Hares," it would be next to an impossibility to expect to receive but a few stings.

At length I started to work, receiving a sting upon my ankle as a warning; it became painful; in a few minutes another "touched" me, another pain. I could not stop; I must work on to the finish now I had started. Sting followed sting in succession, on legs, arms, fingers, neck and face. I imagined what a picture I would present, closed eyes and swollen hands and feet. I worked on, and so did the bees. I could feel the needle-like thrust, but then it did not seem to pain as much, and I at last finished the task. With aching head, slight nausea and vertigo slowly coming on, I left my task with a sigh of relief for what was accomplished, and filled with wonderment as to what my personal appearance would be.

Imagine my astonishment to find merely slightly raised red spots, like little pimples, with the sting in the center, as the result of each and every sting. I must have had something like forty of them on various parts of my body. My clothes were full of them, but they, being so thick, did not allow the stings to penetrate. The dizziness, nausea and headache left me, and "Richard was himself again."

When I again visited my bees, I did not dread the stinging properties any longer, at least not as much so as formerly; and then, and ever since, I have found that when a bee *does* sting me the pain is only sharp for an instant, and there is an absence of the after swelling.

Upon reading up authorities on bee matters, I found that whereas the poison of snakes only affects the circulatory system and can be *swallowed* with impunity, the poison of the bee acts with great power on the organs of digestion as well as that of the circulatory, and produces the nausea, vertigo and headache which I experienced.

I have since been stung many more times than I was at that time, and yet none of the symptoms above referred to have been reproduced. Am I not, therefore, immune to the poison of the honey bee, at least to a certain extent?

All authorities on bee culture state the fact, as a crumb of comfort to novices in bee-keeping, that the poison of a bee will produce less and less effect upon their systems.

"Old bee-keepers," it is said, "like Mithridates, appear almost to thrive upon the poison itself."

Huish speaks of "seeing the bald head of Bonner, a celebrated practical apiarist, covered with stings which seemed to produce upon him no unpleasant effect."

Rev. Mr. Kleine advises beginners "to allow themselves to be stung frequently, assuring them that, in two seasons, their systems will become accustomed to the poison."

If a bee is very angry and elevates that portion of his body containing the sting, a tiny drop of some transparent liquid can often be seen on the point of the sting. This liquid is the poison of the bee-sting. Its taste is sharp and pungent. If it gets into the eyes, as often happens, it has a stinging, acrid feeling, as if, as one writer says, "it might be a compound of cayenne pepper, onion juice and horseradish combined." And when you consider these properties and qualities—especially marked and remembered by one who has experienced them—we cannot think it so very strange that such a substance, introduced into the circulation, produces such exquisite pain. In composition the poison has been shown to be similar to that of the viper and scorpion. Whether it contains fornic acid, as the acid obtained from the ants is called, or not, I do not know. I have never heard that any chemist has given an analysis of it. As the bee feeds upon honey and the pollen of plants, it is undoubtedly a vegetable acid, for it is well known that the poison is much more pungent when bees are working in the fields and accumulating stores largely, than it is when they are at rest in the winter months. As a rule it is during the basswood (American linden) bloom and buckwheat time that we get those severe stings which draw the blood and show a large white spot around the wound.

An old English apiarist advises "a person who has been stung, to catch another bee as speedily as possible, and make it sting in the same spot." Even an enthusiastic disciple of Francis Huber, of Geneva, might hesitate to venture on such a singular remedy.

Lorenzo Lorrain Langstroth, the "father of American apiculture," says: "Allowing a sting to remain until it had discharged all of its poison, I compelled another bee to insert its sting, as nearly as possible, in the same spot. I used no remedies of any kind, and had the satisfaction, in my zeal for new discoveries, of suffering more from pain and swelling than for years before."

Huber has demonstrated that "bees have an exceedingly acute sense of smell, and that unpleasant odors quickly excite their anger." Long before him, Butler stated "that their smelling is excellent, whereby, when they fly aloft in the air, they will quickly perceive anything under them they like, even though it be covered."

They have a special dislike to those whose habits are not neat, and who bear about them a perfume not the least resembling

"Sabean odors
"From the spicy shores of Araby the blest."

Strong perfumes, however pleasant to us, are disagreeable to bees; and Aristotle observes, that they will sting those scented with them. Indeed, I have known persons ignorant of this fact to be severely treated by bees.

On the other hand, some persons, however cleanly, are assaulted by bees as soon as they approach their hives. It is related of a distinguished apiarist that, after a severe attack of fever, he was never able to be on good terms with his bees. That they can readily perceive the slightest differences in smell is apparent from the fact that any number of bees, fed from a common vessel, will be gentle towards each other, while they will assail the first strange bee that alights on the feeder.

To show the virulency of the poison let me quote the following from the writings of an old expert apiarist: "I have known of a horse which, happening to be loose in a bee yard, was attacked by a few bees. In trying to defend himself against them by kicking and rolling he upset one hive and then another; the tens of thousands of bees assailed him, and the poor animal was stung to death before his owner could come to the rescue. I was informed by an eye-witness that although the carcass remained unburied two days neither dogs, crows, buzzards, nor any of the usual scavengers of decaying flesh, attempted to feed upon it, so great was the amount of poison instilled into it by the revengeful bees."

The smell of their own poison produces a very irritating effect upon bees. A small portion of it offered to them on a stick will excite their anger.

Old Butler says: "If you are stung, or any one in the company—yea, though a bee had stricken but your clothes, especially in hot weather—you had best be packing as fast as you can, for the other bees, smelling the rank flavor of the poison, will come about you as thick as hail."

In conclusion let me state that I firmly believe that the bee-keeper becomes inoculated with the poison of the bee, and usually becomes proof, or at least immune, against it, is no more to be doubted than the fact that vaccination is a preventive against smallpox.

In this day of ours, when we see science making rapid strides forward in her various branches, and particularly so in medical sciences, in the time of inoculations as preventive medicine, Pasteur, Koch and others with their serum for hydrophobia, phthisis, diphtheria, etc., would it be surprising if we should hear of the discovery of the use of the virus of the honey bee for the preventive treatment of some disease. Now the healing quality of the venom lies dormant and imprisoned. But who can say how long before science will throw open the doors and deliver it for the benefit of mankind, thus adding a new qualification to this busy and interesting insect.

In this connection it may not be amiss to say to the country physician that the study of the honey bee and the action of its sting may not only be a source of great pleasure but also of profit, as a hive, properly cared for, will produce from fifty to one hundred pounds of honey. There is no expense required, but simply care and a knowledge of the habits of the bee.—*Henry F. Parker, M.D., in Medical Times.*

---

### TREATMENT OF PROLAPSE OF THE RECTUM.*

The treatment of prolapse of the rectum has been extensively written on in the past few years. Treatment of this affection prior to this had been attended with poor results. Of course, allusion is made to prolapse in the adult, as this trouble in the infant is easily managed. VanBuren, after repeated trials of all suggested methods up to the time that he wrote, admitted that none of them afforded relief, or were in any way satisfactory. He therefore devised a method of his own, which was to draw a number of parallel lines half an inch apart, beginning three inches above, and terminating at the anus. Stress must be laid to the fact that these lines must be made deep enough to make good cicatricial tissue. The plan has not proven successful in the hands of the writer.

As long as twelve years ago I suggested the use of the silk ligature in tying off small portions of mucous membrane, just as is done in operating for internal hemorrhoids. In partial prolapse this will be found a good plan. The application of small clamps, left *in situ*, would accomplish the same result. I would object, however, to the plan practiced by some of suturing together the edges and then removing the clamps. Good cicatricial tissue cannot be had by such a procedure.

Gant has suggested a "wire operation" for the relief of prolapse of the rectum, which is done as follows: "Through a posterior median incision extending from the middle of the sacrum to within half an inch of the anus, the coccyx is excised and the rectum freed from its attachments. A fine silver wire mattress, half an inch in width and eighteen inches in length, is then wrapped in spiral fashion around the free portion of the bowel, working from below upward, and fastened in place by a number of fine wires bound around it at intervals of half an inch. The incision is then closed with catgut.

*Read by Joseph M. Mathews, M.D., Louisville, Ky., before the Louisville Surgical Society, Stated Meeting, October 6, 1902.

Dupuytren removed an elliptic fold of integument, including a portion of mucous membrane, at three equidistant points at the anal orifice. This procedure has been practiced several times by the writer, but with *nil* effect. Different operators have modified the operation, but none of them meet the requirement. Gant also suggests a posterior proctoplasty for the cure of the affection, but he admits the great danger of infection by such an operation. Verneuil practices a fixation of the bowel to the sacro-coccygeal curve. Lange makes a posterior median incision from the sacrum to the anus and removes the coccyx. The bowel is then scarified and narrowed by buried sutures that when tied produce a fold or tuck.

The cut edges of the levator ani and sphincter externus are then united, a piece of gauze inserted into the cavity of the wound and the external wound closed around the drain. Iodoform catgut sutures are used. Roberts makes a median incision just below the cervix and breaks up the attachments of the rectum with the finger. The knife is then passed into the bowel, and two deep incisions are made, beginning at a point three inches above the sphincter and passing obliquely downward on either side of the median line through the anus to join the first incision. The triangular piece of tissue, composed of the mucous membrane, muscular coats of the bowel and an inch of the sphincter muscles and the attached skin, is removed. The bleeding vessels are ligated and the rectal portion of the wound closed with sutures of chromicized catgut. A drain is placed in the wound leading to the coccyx and wound closed by deep sutures.

Amputation of the mass has been practiced by a number of surgeons, but has not proven to be satisfactory. It is a hazardous procedure for many reasons, and can never become popular. Such men as Mikulicz, Treves, Kleberg, Fowler and others have originated special ways of amputation, but their success has not been sufficient to cause many to attempt it. A more recent operation has come in much favor both in this country and in Europe. Allusion is made to ventral fixation, which is designated by different men by different names, as proctopexy, sectopexy, sigmoidopexy and colopexy. It occurs to the writer that the last is the most appropriate term. At least it is the colon that is dealt with, and it should receive the distinction in name. It is interesting reading to notice the different plans that are followed in doing the operation. The question is yet mooted as to who first practiced this method. The names mentioned are Herbert Allingham, McLeod, Berg, Cady and others. Any way, it can be safely said that the first operation of the kind was done within the last dozen years. In this country the following named have reported successful results after said operation: Hearne, Boon, Tuttle, Gant, Noble, the writer, and an interesting case is reported by Jacoby, as follows:

*Sigmoidopexy.*—The case reported by Jacoby was one of long standing and severe rectal prolapse. It was completely relieved by a McBurney incision of the left side, incision of the peritoneum, and the sigmoid drawn until all the slack had been taken up and sutured to the abdominal wall with a kangaroo tendon suture carried under the anterior longitudinal band, then carried obliquely across, after which it was again passed under the band. Thus there existed two free ends opposite each other, which were carried through the peritoneum, transversalis, and internal oblique muscles on each side, respectively, giving an indirect figure 8 when the two ends were tied. They were tied so that the opening was nearly closed and the bowel held firmly against the parietal peritoneum and abdominal wall. The remainder of the opening was closed with another kangaroo tendon, and the skin incision brought together with interrupted silkworm-gut sutures. The patient was kept on liquid nourishment for a little over a week and then put on a light diet, which was quickly changed to the full diet. He asked for discharge twenty days after operation. Jacoby thinks the figure 8 suture that is employed is worthy of consideration because it brings into close and continuous contact the two serous surfaces, as well as the abdominal wall and intestine, and is better able to sustain the weight and tension.

Noble said in reporting his case: "I desire to advocate a simple method of operation which promises well in the treatment of intractable cases, and to report two cases in which this operation has been performed. The operation which I would propose is to open the peritoneal cavity by an incision made through the left rectus muscle slightly below the promontory of the sacrum, to search for the sigmoid or for the rectum and to make traction upon it until it is inverted and until 'the slack' has been taken up. The point at which the lower portion of the rectum will come in contact with the abdominal wall on slight tension should be determined, and this point attached to the abdominal wall by three or more fine silk sutures. The sutures should be passed so as to include a portion of the rectus muscle, and should pass under the anterior longitudinal band of the rectum. In this way the bowel can be firmly attached to the abdominal wall with the least danger of penetrating its lumen and with the greatest prospect of permanent attachment. The abdominal wall should then be closed by the tier method."

In April, 1899, I operated on a very notable case of prolapse of the rectum by this method, assisted by Dr. Ap Morgan Vance, who gave me many valuable suggestions. When it occurred to me to open the abdomen in this case, it was simply carrying out a suggestion that I had made to Dr. Wm. L. Rodman ten years before, as he testified when I read a report of the operation before the Chicago Medical Society by invitation. And at the time the operation was done I had never seen in print or heard that it had been done. Afterward I saw that Mr. Carlyou had said that a report of three such operations had been made. During the discussion of my paper before the Chicago Medical Society, Dr. J. B. Murphy said that priority should be given me, as any operation that had ever been done was for conditions far less simple than had been encountered by me, as an elongated mesentery causing a prolapsed condition of the upper into the lower rectum.

I will not consume your time by reporting either the history of the case or the operation, as it has already been reported, and has appeared in the *Journal of the American Medical Association.* I desire simply to say that the case was of thirty-five years' standing; that the prolapse was as large as an ordinary head; that it contained all coats of the bowel, the peritoneum and the bladder.

The abdomen was opened and the colon anchored to the muscular abdominal wall by chromicized catgut sutures. I have written this paper, however, to report another method of operating for this very formidable affection. A patient was referred to me recently from one of the Westerm States, who had a prolapse of the rectum which included all the coats of the bowel and was the size of a large fist. After preparation he was chloroformed, and a circular incision was made around the protruding mass just without the anus. A second incision was then made around the bowel near the distal end of the tumor, when the "cuff" between the two incisions was carefully dissected and removed. The divided ends of the gut were then drawn together, closely approximated, and sutured. Dr. H. H. Grant, who assisted me, suggested the cutting out of two elliptical pieces in order to get closer approximation, which was done. Of course in such a dissection much of the submucosa was included. The operation produced a perfect cure.

Duret, in his operation, dissects out an elliptical flap of mucous membrane from the anteriorand posterior surfaces of the prolapsed gut, from the summit to the base, and then folds the muscular walls in by buried silk sutures and superficial ones to approximate the edges of mucous membrane. Delorme's operation is very like the one of my own. He dissects the mucous membrane from the prolapse, denuding the entire rectum, and sutures to the mucocutaneous border, invaginating the gut above the line of sutures. In my opinion the dissection of the mucous membrane alone will not accomplish a permanent result, but that the deeper tissues must be included, as was done in my case.

The Dieffenbach-Roberts operation consists in the removal of a section of the gut at its posterior commissure extending two inches upward—the entire thickness of the intestine, with the sphincter muscle, is removed. I cannot think that this is necessary, for a less amount of surgery will accomplish just as much and the sphincter muscle be kept intact. Lange infolds the rectal ampulla from the outside. This is a different operation, and the results are no better than others. Peters operates upon the anterior wall by making an abdominal incision in the median line; the prolapse is drawn upward by pulling the sigmoid; the anterior wall of the gut is infolded by Lembert sutures; the ends are left long and pass through the muscular layer of the abdominal wall, forming a sling to support the rectum. Fowler first practiced suspension of the rectum by sutures carried around the coccyx, and Tuttle has elaborated the plan.

So it can be seen that many operations have been devised, modified and elaborated for the cure or betterment of this very formidable affection, but the writer believes that in colopexy we have the best plan yet offered for the cure of a prolapse of any dimension. In the operation no injury is done the gut itself; the sphincter muscles are not disturbed, and the danger is less than in the other operations. In a prolapse of less degree, the operation of Delorme or the one suggested by the writer, will meet the demand satisfactorily.—*Louisville Journal of Medicine and Surgery.*

---

### VOMITING IN PREGNANCY.

In early pregnancy, morning vomiting which usually follows taking of food is a normal condition. This I shall designate the benign form.

If by its persistence and increasing frequency it prevents proper alimentation, causes great emaciation and weakness, it becomes what may well be called the grave form.

And if it proves to be rebellious to all treatment short of removing from the uterus the *fons et origo* of the trouble, this condition I shall designate the uncontrollable form. The conditions which enter into its etiology are manifold.

We see cases, without discoverable lesion, in which, as soon as the impregnated ovum finds lodgment in the uterus or tube, vomiting begins. It may be a few days or it may be a few weeks after impregnation. This nausea and vomiting may continue despite all our efforts to correct it until the uterus is emptied, when immediate relief follows.

It seems probable that the increased vascular supply to the uterus and the decreased vascular supply to the brain, together with its concomitant nervous disturbance, afford us the only explanation of this condition in the present state of our knowledge.

If we can find a malposition, erosion or other diseased condition of the uterus or of its neighboring organs, we think we have here the complicating conditions towards which we should especially direct our treatment. Real pathological changes in the cervix will likely preclude the possibility of the completion of gestation. The severity of the vomiting, however, is usually out of proportion to the discoverable lesion or lesions, as the case may be.

I have seen a small ulcer situated on the os, produce for a week almost continuous nausea and vomiting, which was immediately relieved after cauterization. My experience has been that women of a nervous temperament, and those who vomit with facility under other conditions, have most trouble with the stomach during pregnancy. And while vomiting of pregnancy is usually of a reflex nature, the stimulus from the growing ovum exciting through the uterine plexus the center of which controls vomiting; nevertheless, microscopic changes of the stomach are sometimes found in cases resulting fatally from vomiting of pregnancy.

The lesions of the stomach may have been preexistent; or the lesions may have been produced by the continued stomach derangement. In vomiting of pregnancy there seems to be a sort of gravidic autointoxication which acts mechanically by changes taking place in the uterus, and reflexly through the vomiting center.

All vomiting presupposes the existence of a vomiting center, which is situated in the floor of the fourth ventricle.

Excitation of this center causes an excitation of the parietal, phrenic, and pneumogastric nerves, which supply the abdominal muscles, the diaphragm, and the stomach and esophagus, which muscles are directly concerned in the act of vomiting. The excitation of this center may be of central organ, as by a blood-clot or neoplasm.

It may be due to the presence in the blood ot some toxic emetic substance, or to some local excitation remote from vomiting center.

Among the causes of vomiting which continue to be of especial interest are the well known, but not well understood, psychic influences. Simply the thought of loathsome objects in those persons who vomit with facility will produce it. I have now under my care a lady suffering from uterine cancer, who frequently vomits at the thought of taking food. This is one of the many conditions we meet, which impress upon us the thought that we are fearfully and wonderfully made.

In the benign form of vomiting of pregnancy, attention to diet, proper physical exercise, and where there is pyrosis alkaline treatment is usually all that is needed. Sometimes anti-emetics, such as cerium oxalate and bismuth subnitrate, are valuable. In the grave form vomiting persists and increases in frequency until the patient is greatly emaciated and weakened.

In many of these cases the interdiction of solid food, the use of anti-emetic medicines and such other treatment as may be indicated, such as the correction of displacements, etc., together with regulation of the bowels, and last but not least, the regulation of the patient's physical exercise and the maintenance as far as possible of cheerful surroundings, will bring about relief. A voyage, the satisfaction of a desire for certain articles of diet, etc., may prove to be more efficacious than medical treatment. In obstinate cases of this form slight dilatation of the cervix may cure, as was discovered by Copeman, who, in an unsuccessful attempt at emptying the uterus by dilatation, found his patient was relieved by this dilatation alone.

In other cases the reclining posture, with elevation of the hips, controls vomiting. The different forms of pessaries may relieve certain cases. Lavage of the stomach has proven of great value in some cases, but of this treatment I can say nothing from experience. However, I believe it will grow in favor with the medical profession. For what is more reasonable than to give rest to a weakened or offended organ, and if need be, to saddle its work upon some more tractable one. The rectum may very nobly perform this vicarious function for the irritable and rebellious stomach.

If all these means fail the case falls in the category of the uncontrollable form. We should not delay too long the treatment necessary in this form, as delay may result disastrously. If need be, we should unhesitatingly shoulder our manifest responsibility, obtain consultation, and after due consideration of the case do our duty by removing the offending cause.

The first case I wish to report is that of a young woman, twenty-one years of age, of slight build, delicate appearance, and who has suffered from dysmenorrhea since the first appearance of menstruation. At her menstrual periods she had always suffered with pain in the left side, as well as in the region of the uterus. This condition often led to syncope. She had medical treatment but without any benefit up to the time of her marriage. After this time she came under my care. Upon examination I found the uterus retroflexed and slightly twisted to the left. I advised local treatment, but this advice was not favorably considered, and a trial of gelsemium was resorted to for one month. This treatment rendered the next menstrual period much more tolerable; but after another month's trial of this treatment it was found necessary to resort to instrumental treatment, which was dilatation and curettage. The two periods subsequent to this last treatment were passed without pain; these being the only periods ever passed without pain since the beginning of menstruation. Before the third period after curettage, the patient conceived, and after the first four weeks of pregnancy was troubled with nausea and vomiting at all times of the day.

Alkaline treatment was not of much benefit, but a combination of

| | | |
|---|---|---|
| ℞ | Cocain muriate | 1/8 gr. |
| | Cerium oxalate | 5 grs. |
| | Bismuth subnitrate | 10 grs. |
| | Elix. simp | 1 dr. |
| M. | | |

afforded marked relief. Mellin's Food was well tolerated. The bowels were regulated admirably by the syrup cascara aromatic. At term the delivery of a vigorous eight-pound boy was accomplished without accident.

The second case, a well developed, large woman, 28 years of age and pregnant for the first time. She had brown spots on skin before and after conception. Vomiting began after the fourth week. Many remedies were used but without any apparent benefit. The vomiting continued, but not severe enough to prevent proper alimentation, throughout the entire period of gestation. This patient found that whisky helped her and resorted to it daily. After delivery, the babe was found to be extremely fond of whisky. This desire was probably engendered while *in utero*. If such was the case it should teach us to proscribe the use of strong stimulants except under extraordinary conditions.

Case third, a delicate woman, aged 46, pregnant for the eighth time. Was not troubled much during the first few months, but at the seventh month it became necessary to resort to an exclusively liquid diet. Milk with Maltine was at times all that could be retained. The anti-emetics at times allayed the stomachic irritation, and at other times were valueless. About the middle of the eighth month bronchitis developed, with severe pain in the shoulder. Owing to the irritable condition of the stomach heroin was selected to allay pain and cough. It was well tolerated by the stomach. I have used heroin in doses beginning with 1-10 grs. and increased to 1/3 grs., with excellent effect in a case of uterine cancer in which neither morphia nor codein could be tolerated by the stomach.

Case fourth, wife of a physician, 22 years of age; second pregnancy. There was some vomiting before the sixth week, but at this time it became more persistent, and after several weeks the patient became very weak and emaciated. At this time I was called in consultation. No drug seemed to have had any effect on the vomiting. Finally, after consultation by three physicians, dilatation of the cervix was tried, without any effect. After a few days it was deemed necessary to empty the uterus. Dilatation and curettage was resorted to. The fetus removed was about four inches in length. After this, under strict dietary restrictions, there was no further trouble with the stomach, and the patient made an uninterrupted recovery.—*O. M. Bourland, M.D., Van Buren, Ark., in Medical Times.*

---

### A NOTABLE IMPROVEMENT IN THE THERAPY OF TYPHOID FEVER.

The recent discovery, by Duval and Bassett, of the presence of the bacillus dysenteriæ (Shiga) in forty cases of infantile summer diarrhea awakens renewed interest in the subject of intestinal antisepsis. But a few months have elapsed since Drs. P. C. Freer and F. G. Novy, of the University of Michigan, demonstrated the enormous germicidal power of benzoyl-acetyl-peroxide, more familiarly known as Acetozone. Although the preliminary reports of these investigators were of necessity based upon results of laboratory experiments, their expectations are already being realized in clinical work, in the treatment of typhoid fever particularly.

In the city of Chicago, where a large number of cases of typhoid have been reported, Acetozone has been used exclusively in the treatment of about 300 of them. The consensus of opinion is that it causes the temperature to decline earlier than usual in the course of the disease, and it ameliorates the mental and physical condition of the patient, in all probability by controlling the toxemia.

Two Chicago practitioners, I. A. Abt, M.D., and E. Lackner, M.D., have thus far reported (*Therapeutic Gazette*, October, 1902) forty cases of typhoid, in children, treated with Acetozone, with but two deaths, a mortality of 5 per cent. One of the patients that died succumbed to pneumonia and pulmonary edema, the other to great pyrexia on the fifth day. Stupor and tympanites were almost entirely absent in all the cases; the characteristic typhoid fetor of the stools was markedly diminished, and the hemorrhage occurred but twice, and in the same case. The average duration of the febrile period, in 37 cases, after beginning Acetozone treatment, was 13½ days. The drug did not seem to act upon the heart or respiratory apparatus.

Early this year Eugene Wasdin, M.D., of the U. S. Marine Hospital Service, Buffalo, N. Y., reported 27 cases (*American Medicine*, February 8, 1902) of typhoid fever, 24 of which were treated with Acetozone, all of the patients recovering. The writer says: "Its application in typhoid fever has been followed by very happy results; its use has been directed to the destruction of the germ in its primary lung colony and also in its secondary intestinal colony, and it has been used by hypodermoclysis to combat terminal expressions, with the result that in 24 cases the disease has been limited almost entirely to the expression of intoxication from the primary focus, the intestinal symptoms remaining entirely in abeyance, and the disease has been shorn of many of its most disagreeable features."

In a second paper, which appeared in the *Therapeutic Gazette* for May 15, 1902, the same writer states that his patients were given from 1,500 to 2,000 cc. of the aqueous solution of Acetozone daily. The diet was milk, diluted with the same solution. The first influence of the drug is observed in the increased secretion of urine. That this is not due wholly to the ingestion of large quantities of water necessitated by the use of the saturated solution is evident from the author's assertion that the same result was observed when Acetozone was administered in capsules. The second influence to which attention is directed is the very pronounced decrease of the odor of the stools, while plate cultures from the dejecta showed comparatively few germs.

The deodorant and diuretic effects of Acetozone were also observed by G. H. Westinghouse, M.D., of Buffalo (*Buffalo Medical Journal*, August, 1902) who used it in seven cases. This observer remarks that with the increased flow of urine "a corresponding reduction of typhoid symptoms followed, and tympanites and delirium disappeared." It should be remarked that the diagnosis in all these cases, as well as in most of those reported by the Chicago physicians, was confirmed by Widal's reaction and Ehrlich's test, and in some a blood-count was resorted to. Westinghouse concludes his paper by saying that "Acetozone, as an intestinal antiseptic, is unequalled by anything I have ever employed. A complete subsidence of all the bowel symptoms followed in every case of typhoid within a few days after beginning its use. The application of the antiseptic consisting, in most cases, in simply allowing the patient to drink the saturated aqueous solution *ad libitum;* or, in other words, substituting this solution for all other liquids, and urging the patient to partake of it freely when the natural craving was not sufficient to insure the consumption of considerable quantities."

